# Publication of inspection frameworks: a qualitative study exploring the impact on quality improvement and regulation in three healthcare settings

**DOI:** 10.1136/bmjqs-2020-011337

**Published:** 2020-12-02

**Authors:** Jan-Willem Weenink, Iris Wallenburg, Ian Leistikow, Roland A Bal

**Affiliations:** 1 Erasmus School of Health Policy & Management, Erasmus University Rotterdam, Rotterdam, Zuid-Holland, The Netherlands; 2 Dutch Health and Youth Care Inspectorate, Utrecht, The Netherlands

**Keywords:** healthcare quality improvement, health policy, patient safety

## Abstract

**Background:**

The Dutch healthcare inspectorate publishes its inspection frameworks to inform both the public and healthcare providers about regulatory procedures and in the hope that publication will motivate healthcare providers to improve quality and comply with standards. This study explores the consequences of publishing these frameworks for the regulation of quality and safety in healthcare.

**Methods:**

We selected recently published inspection frameworks used in three healthcare settings: nursing home care, dental care and hospital care. We conducted 37 interviews with 39 respondents (healthcare professionals, managers, quality officers, policy advisers and inspectors) and explored their awareness of and experiences with these frameworks. We held a group interview with three inspectors to reflect on our findings. All data underwent thematic content analysis.

**Results:**

We found that the institutional infrastructure of a sector plays an important role in how an inspection framework is used after publication; particularly the presence and maturity of quality improvement work in the sector and the inspectorate’s grip on a sector matter. Respondents mentioned differences in framework use in organisational contexts, particularly relating to scale. In some organisations, the framework served as an accountability mechanism to check if quality meets basic standards, while in other organisations professionals adopted it to stimulate discussion and learning across teams.

**Conclusion:**

Publication of inspection frameworks might result in quality improvement work, and in particular contexts could be used as a regulatory strategy to target quality improvement in a healthcare sector. For this, it is important that regulators consider the capabilities and possibilities for learning and improving within a sector.

## Introduction

In most healthcare systems, healthcare regulators have an important responsibility to monitor the quality of care.^1 2^ The expectation is that inspections will impact positively on the quality and safety of care, although there is little direct evidence of the effectiveness of external inspections on quality of care within organisations.^3–6^ A recent report from the King’s Fund explored the impact of regulation and identified ways in which regulation might impact provider performance before, during and after inspection: for example, healthcare providers might anticipate an upcoming inspection and make changes in practice, become aware of specific quality and safety concerns raised during inspections, and might initiate broader organisational developments, reflection and analysis following the inspection.^7^ Such reactions might positively affect healthcare quality but can also have unintended effects. Previous research into the impact of National Health Service inspections in England showed that healthcare providers sometimes game inspections; inspection scores are elevated around the time of an announced inspection without producing a long-lasting effect.^8^ Strategic behaviour by healthcare providers might also result in the negligence of quality aspects that are not included in these inspections.^9^ In this study, we focus on the impact before inspection, that is, on the impact of the inspection frameworks regulators use.

In the Netherlands, the Dutch Health and Youth Care Inspectorate is becoming increasingly transparent about their working procedures. These include inspection frameworks, which list the legislation and professional standards on which healthcare providers are assessed during inspections. Regulators in other countries, such as the Care Quality Commission in England, also publish inspection frameworks.^10^ By publishing these frameworks, the Dutch inspectorate informs the public and providers about regulatory procedures. At the same time, the inspectorate hopes that the framework publication might serve as an incentive for healthcare providers to improve quality and comply with standards, regardless of whether the healthcare provider will be inspected or not. Hence framework publication is intended to impact quality improvement in all healthcare providers, not just those inspected. As the capacity for inspections is limited and it is hard to prioritise inspections based on surveillance data of healthcare providers, it seems important for regulators to come up with additional strategies to target quality improvement in healthcare sectors.^11 12^

Little is known, however, about the consequences of making inspection frameworks public, and the extent to which healthcare providers are aware of and use these frameworks for quality improvement. In this study, we explore the consequences of publication of inspection frameworks, with the objective of gaining insight into their function in regulating quality and safety in healthcare.

## Methods

### Setting

Our study focuses on regulation in the Netherlands. In the Dutch healthcare system, healthcare professions are responsible for setting professional standards. The government develops general legislation for quality and safety of care, often consulting professional and provider associations.^13^ The role of the inspectorate is to supervise quality and safety of both healthcare organisations and individual healthcare professionals, by checking whether organisations and professionals comply with professional standards and legislation.^14^ The inspectorate uses two approaches: incident-based supervision following incidents and complaints in healthcare, and risk-based supervision focusing on specific themes or type of providers. The inspectorate uses the available standards and legislation to design inspection frameworks that are used during inspections. These frameworks focus on specific themes or risk areas, such as radiology in dental care or operating theatre hygiene, and multiple frameworks are used within sectors. In general, the inspectorate conducts their inspections unannounced. They do announce forthcoming inspection rounds on specific themes, indicating that they will make a number of unannounced visits in the upcoming months. The inspectorate drafts a report for each organisation it has inspected, as well as an overall report on the theme. These reports are also published. The expectation of the inspectorate is that inspections will drive compliance and stimulate quality improvement as non-compliance is pointed out to providers, and that subsequent reports of inspections and the overall report will inform and motivate a sector to further improve on the inspected themes.

### Study design

As we were interested in the underlying mechanisms and processes of inspection frameworks post publication, we adopted an exploratory qualitative approach. We opted for maximum variety, and in consultation with the inspectorate selected three frameworks that differed in context, design and method of publication.^15^ These were frameworks on quality of nursing home care, radiology in dental care and infection prevention in hospital care. All were published in 2017 and subsequently used for inspecting a select number of healthcare providers. Table 1 describes the three frameworks.

**Table 1:** The three inspection frameworks

	Quality of nursing home care	Radiology in dental care	Infection prevention in hospitals
Target providers	±700 nursing care providers with ±2300 nursing home locations.*^29^	8265 dental practices: 3990 with 1 employee, 1405 with 2 employees, 1460 with 3–5 employees, 1405 with >5 employees.^30^ 6% of practices belong to a dental chain of practices. In 2017, the three largest chains contained 47–80 practices.^31^	71 general and 8 academic hospitals.^32^
Focus of framework	Three themes: (1) person-oriented care, (2) professional expertise, and (3) governance of quality and safety.	Three themes: (1) safety, (2) expertise and (3) quality.	Six themes: (1) general measures, (2) cleaning and disinfection, (3) isolation measures, (4) risk inventory, (5) antibiotics use, and (6) quality and protocols.
Design of framework	Describes (1) the relevant standards, (2) source of these standards (eg, which guideline or law) and (3) explanations for what the inspectorate might look at for each standard.	Describes (1) the relevant standards and (2) the source of the standards.	Mentions the considered guidelines and legislation. The inspectorate has a separate instrument for inspections (not public) that describes the specific aspects under assessment.
Assessment of standard in framework	Completely positive. Mostly positive. Mostly negative. Completely negative.	Positive. Negative.	Positive. Negative.

*There is no available registration of nursing care providers. The inspectorate does not know all of the providers because new providers regularly enter the market, existing providers merge and other providers go into administration.

### Data collection and analysis

We used purposive sampling to conduct semistructured interviews with managers, quality officers and healthcare professionals from healthcare organisations, policy advisers from professional associations, and healthcare inspectors.^16^ The topic list for our interviews was based on a literature scan conducted for the research project and included (1) awareness of and attitude to the inspection framework, (2) how the framework was used in practice, (3) its influence on compliance to norms and quality work, and (4) interaction between providers, professionals and associations on the one hand and the inspectorate and inspectors on the other (online supplemental appendix 1).

10.1136/bmjqs-2020-011337.supp1 Supplementary data

We held 37 interviews with 39 respondents. table 2 gives an overview of interviewees from each setting. Two researchers experienced in conducting qualitative research (J-WW, IW) led the interviews, which were recorded and transcribed verbatim. All participants provided verbal consent before the interviews, which were held between June and September 2018. Two researchers (J-WW, IW) used thematic analysis to identify overarching patterns in our data.^17^ Throughout the analytical process the coding template was adapted and a third researcher (RAB) was consulted to discuss and review identified themes. After analysing all interviews, the first two researchers held a group interview with one inspector from each setting. The goal of this group interview was to reflect on our findings with the three inspectors and gain a better understanding of the similarities and differences between settings. The group interview took place in November 2018 and was structured and analysed around the identified themes. All transcripts were coded using Atlas.ti V.8.

**Table 2:** Overview of interviewees for each inspection framework

Quality of nursing home care (n=18)	Radiology in dental care (n=10)	Infection prevention in hospitals (n=11)
1 healthcare executive. 2 managers of care and well-being. 2 location managers. 2 quality officers. 1 team leader. 2 nurses. 1 medical specialist in elderly care. 1 adviser of a consultancy firm that organises nursing home audits. 4 policy advisers from professional associations. 2 inspectors.	3 dentists. 3 dentists who act as policy advisers for a professional association or chain of dental care practices. 2 policy advisers from a professional association and chain of dental care practices. 2 inspectors.	3 medical microbiologists. 2 hospital hygienists. 3 policy advisers from hospital or medical specialist associations. 3 inspectors.

## Results

The main themes that emerged from our data relate to the development of inspection frameworks, the uptake of a framework once published, how the inspection framework contributes to quality improvement in practice, and experiences with the frameworks during inspections.

### Development of inspection frameworks

Three issues seem crucial to the inspectorate in designing inspection frameworks. First, the framework must be grounded in the law or professional standards, to ensure it is legally watertight. Second, the framework must have social legitimacy. The inspectorate consults professional associations or experts put forward by professional associations during the framework development. This consultation serves as a check on the selected and prioritised standards and enhances the legitimacy of the framework. Third, a framework should be pragmatic and should include important current quality issues. The inspectorate prioritises standards in their frameworks. This means that a framework is a selection of professional standards and not a complete overview of all relevant standards.

We’re all practical. We know it’s our goal to do an inspection in one day. You could also make a framework that will take you four days. So we try to develop a manageable framework and that means it won’t include many things. (R17: inspector, nursing home care)

The assessment method in the frameworks differs. Some contain standards that are assessed dichotomously (positive/negative), while others include standards that leave room for interpretation and nuance in applying a four-point assessment scale. Departments consciously make room for interpretation and nuance when developing frameworks. For nursing home care, the decision to use an assessment scale came from the aim to stimulate the organisational learning process of organisations while keeping its context in mind.

We use a four-point scale so that we can show nuances and [we] also incorporate the nursing home’s context in our judgement. So maybe two providers have the same description for a standard, but for the one it leads to a positive judgement and for the other, a negative one. It demands a lot from inspectors, to do that properly. [It takes] a lot of coordination and discussion. (R19: inspector, nursing home care)

The framework allows for responsiveness to the needs and capabilities of a healthcare organisation. In contrast, the dental care framework was based on pragmatic choices due to time and capacity constraints, resulting in a framework that is quick and easy to check off.

The development of a framework continues after it is published and some inspectors indicated that work on the framework is never entirely finished. For infection prevention in hospitals, rounds with earlier versions of the framework produced criticism from medical specialists.

After the first round of inspections, there were lots of comments about the framework and the standards it included. After that, we held a discussion group with all relevant stakeholders, like professional associations. There was lots of discussion on which standards should be left out of the framework for the next round and about which interpretations of standards should be adjusted. (R38: inspector, hospital care)

According to the physicians, the inspectorate focused too much on irrelevant process indicators (eg, the ‘wetness of cleaning wipes’), while attention should be drawn to outcomes ‘to have real impact’. The inspectorate took this input into account and moved away from a focus on process indicators, yet did not choose to focus on outcome indicators entirely as was preferred by the physicians. Instead, it decided to also focus on the overarching governance structure of hygiene in a subsequent version of the framework, forcing hospital management to take responsibility for hygiene and sustainable antibiotics use in their organisation.

### Uptake of inspection frameworks after publication

Following publication on the website of the inspectorate, the frameworks are primarily taken up by policy advisers from professional associations and quality officers in organisations. Healthcare professionals sometimes take notice of the frameworks through newsletters from their profession or their organisation, but quickly seem to forget about them.

They [the inspectorate] seem to think that we’re always on the computer searching for what’s new, if there’s anything new on their website. I don’t have time for that. (R20: dentist)

The extent to which a framework is adopted seems to depend on organisational characteristics. Larger organisations, such as nursing homes, hospitals and chains of dental practices, employ quality staff who closely monitor the inspectorate’s website and translate the frameworks and inspection reports to their workplace. Smaller organisations or independent providers do not do this, as they often do not have people specifically tasked to do so.

I see this in the larger chains. They have quality officers or other people on it who optimize a practice or their chain according to such a checklist. (R25: dentist/policy adviser)

Additionally, differences between sectors related to institutional infrastructure seem to impact the uptake of the framework. As respondents indicated, working on quality improvement is a given in hospital care and increasingly so in nursing home care too. In dental care though, quality improvement is in its early days (eg, a guideline development centre was initiated only recently) and as yet has no prominent place in dentists’ work. Furthermore, the sector has two professional associations who are in constant competition. The inspectorate’s grip on a sector also plays an important role. What happens to the framework seems to depend on the possible consequences that healthcare providers expect in the event of non-compliance with the standards from the framework. In dental care, the consequences seem to contribute little to compliance with the standards. Dentists do not consider the chance of an inspection visit as imminent, as inspectors realise too:

They still have this idea of ‘well, there are 6000 dental practices, what’s the chance that I’ll get caught?’ (R29: inspector, dental care)

Managers in nursing homes, however, realise that the inspectorate will pay a visit and prepare their organisation for this eventuality:

We were just discussing the inspection framework in-house, because we have this feeling that the inspectorate might visit one of our nursing home locations soon. (R1: location manager, nursing home)

The ways that a sector is structured—big or small organisations, existence of guideline committees, organisation of sectoral interests—as well as its relation with the inspectorate thus seem to matter in whether and how inspection frameworks are spread and translated to organisational policies. The presence of developed relations, both between the inspectorate and the sector and between the organisations within a sector, enhances the use of inspection frameworks as quality instruments.

### How inspection frameworks give input to quality improvement

In nursing and hospital care, inspection frameworks seem to put certain quality themes on the agenda; resources and attention are allocated to these themes. How organisations do this, however, differs substantially. In hospital care, the studied framework gave microbiologists a compelling argument to convince hospital management to invest in infection prevention.

After the first inspection round with the framework, microbiologists said that they were really glad that we [the inspectorate] were pushing the topic of infection prevention, because it’s really put infection prevention at the top of the hospital management agenda. (R37: inspector, hospital care)

In elderly care, the framework is used to motivate input for organising internal audits, but organisations manage this differently. This is partly due to ownership. In some organisations, managers and quality officers use an inspection framework to obtain a grip on a department:

The purpose of internal audits? A few times a year we want an independent check of a department, just to see how our care gets assessed from the perspective of the inspectorate. (R4: healthcare manager, nursing home)

Other nursing home organisations give the framework to professionals to chart quality, particularly to discuss how quality can be further improved. These assessments aim to stimulate dialogue between professionals and across departments on what good quality entails and how to organise it. Inspectors acknowledge the varying uses of the inspection framework, either as a checklist or as a reflexive tool, and are aware of its implications for how organisations approach quality improvement work.

I do get signals that consultancy agencies are offering to make practices or organisations inspection-proof. You come across all these checklists, our inspection framework and other things they have done themselves. They check off the lists and think ‘as long as I meet the inspection framework, I’m safe. I don’t have to think anymore’. (R29: inspector, dental care)

Although the inspection framework for elderly care was designed to leave space for organisations to design their own quality policies (within the bounds set by the framework), and to encourage reflection, some consultancy agencies hired by nursing homes still create their own checklists. Inspectors do not necessarily see this as a bad thing, as it does put these quality themes on the agenda of the organisation and generates action. Additionally, quality officers said that published reports of inspections done in other organisations provide more valuable information than found in the inspection frameworks. Compared with the inspection frameworks, inspection reports show how the inspectorate interprets and assesses standards in the inspection framework, providing insight into the inspectorate’s actual expectations.

### Experiences with inspection frameworks during inspections

Inspectors said that they did not notice substantive differences in their work of inspecting healthcare providers after the publication of the frameworks. They still have enough leeway to consider situated aspects that are not included in the framework. Some inspectors mentioned that healthcare providers sometimes have the printed framework at hand during an inspection. This makes the conversation a bit easier, yet it is no guarantee that either the quality aspects in the framework or the overall quality will be enough.

In one practice, when we came in, the printed framework was on top of their folder. That’s perfect, I thought, yet it turned out that not everything complied with the standards. (R28: inspector, dental care)

Inspectors mentioned that both the inspection and the writing up of its report take a substantial amount of time. The duration is impacted by the design of an inspection framework. In elderly care, the focus is on practice (‘observing instead of checking boxes’). This means that the inspector uses information from various sources. They observe, listen, ask questions, and consult protocols and medical records through the Short Observational Framework for Inspection.^18^ Prior to the adoption of this approach, inspection frameworks for elderly care were designed to check off shortcomings. This new approach, consciously made to stimulate learning in healthcare providers, has not only led to lengthier inspections but also to more extensive and frequent discussions and consultations between inspectors when writing an inspection report. These discussions are to ensure consistency across inspection reports when interpreting open norms. This is deemed important as both inspection frameworks and reports become public, and so the inspectorate can be held accountable for potential inconsistencies in interpretations. Respondents that were inspected with the new ‘opening up’ approach were much more positive, as it offers the opportunity to explain such things as why things are arranged in a certain way. The inspectors said that they feel healthcare providers appreciate their new approach.

The inspection we did in 2016 was a big improvement. They were really open, wanted to see things and not just check off shortcomings straight away. And last year, the observation was a very pleasant experience. (R5: quality officer, nursing home)

In all settings, respondents mentioned that during an inspection, they want to be able to explain to the inspectors why things are organised in a certain manner. According to respondents in nursing care, the framework design, with its open assessment of standards, enables such explanation. Conversely, in dental care, inspectors use an iPad to check boxes (positive or negative), which speeds up the visit but provides far fewer opportunities for explanation as the organisation either does or does not comply. Interviewees reported that explaining why things are organised in a certain way does not lead to a different assessment.

## Discussion

We explored the consequences of publication of inspection frameworks in three healthcare settings with the objective of enhancing our understanding of the functioning of inspection frameworks in the regulation of quality and safety. Our findings show that the impact of publication differs between and within sectors and primarily depends on the institutional infrastructure and the relation of the inspectorate with a sector. We also found that inspection frameworks were used for quality improvement in several ways. After a methodological reflection, we elaborate on these findings and conclude with how these insights could serve as input for improving the regulation of healthcare.

### Strengths and limitations

A strength of our study is the inclusion of a variety of sectors, type of inspection frameworks and respondents within the studied settings. As little is known about if and how inspection frameworks are used by providers, this offers a relevant exploration of experiences with and uses of inspection frameworks. The study also has some limitations. First, we purposively sampled a selection of professionals and organisations from each sector and were not able to include all different types of professionals and providers within those sectors. Second, the variety in included sectors and organisations provides insight into the different consequences of publication and uses of the framework, yet caution is required in generalising these findings across and within sectors. Further research is needed to determine which occur when and where, also taking the national context of healthcare regulation into account. Third, we were not able to include dentists that were inspected with the inspection framework and as such only have data from the inspectors’ side on experiences during inspections.

### The impact of the inspection framework depends on the institutional infrastructure

We found that the institutional infrastructure of a sector plays an important role in what happens with an inspection framework after publication, in particular the presence and maturity of quality improvement work in a sector, organisational characteristics within a sector and the inspectorate’s grip on a sector. Previous studies have highlighted the importance of institutionalising quality improvement and patient safety practices in health systems.^19 20^

Different motivations can play a role in whether organisations or professionals comply with standards. Some might be motivated by the possible consequences of (non-)compliance and some might feel an intrinsic obligation to do good.^21^ In our study, the former appears to play an important role. In nursing home care, the general feeling was that the inspectorate will come pay a visit eventually and that consequences could be dire if things were not in order. In dental care, the inspectorate’s grip on the sector is low and dentists did not perceive an inspection as a real ‘threat’, and as such possible consequences of non-compliance did not provide any motivation for quality improvement. This seems to suggest that some providers in nursing care are particularly occupied with ‘looking good’ instead of ‘doing good’; conversely dentists did not seem to be occupied with looking good as the inspectorate does not have a major influence on their work. On the one hand the focus on ‘looking good’ leads to compliance with standards as is the regulator’s goal, yet on the other hand it could lead to ‘hitting the target and missing the point’.^9^ It might additionally deflect intrinsic motivations and reflections on how to organise quality. Especially in our case of nursing care that is unfortunate, as the framework aimed to stimulate organisational learning processes. These findings add to previous studies on how providers might anticipate inspections to look good, by showing how providers might also use documents and procedures of regulators to anticipate the possibility of an inspection in the near future.^7 8^

### Inspection frameworks may act as a lever for quality improvement

Our findings show that the publication of an inspection framework may work as a lever for quality improvement in several ways.^22^ At an organisational level, the framework might be used by professionals to put certain quality themes on the agenda of management, highlighting the coercive use of external regulatory frameworks to drive quality improvement.^13 23^ Second, the framework might be used as a diagnostic tool by management to determine quality of care within their organisation and identify what quality issues require improvement. An accountability mechanism could serve as a good start to get basic quality in place, as a reflective approach requires a broader learning capability of the organisation.^24^ Third, the framework might enable discussion about what quality entails and as such act as a boundary object in knowledge interaction and sharing of meaning.^25^ This can happen between inspectors and professionals during an inspection, and between professionals (and others) when using the framework to audit other departments to provide feedback and discuss learning opportunities.^26^ Finally, at a sectoral level the framework might facilitate an ongoing relation between the inspectorate and the professional and provider associations in which a continuous discussion ensues about what quality entails and how to adequately monitor this, as observed with previous versions of the infection prevention framework in our study. Additional to these levers for quality improvement, another important finding emerged that provides insight into the functioning of inspection frameworks. Quality officers indicated that they not only use the inspection frameworks, but also analyse inspection reports, as these show the inspectorate’s interpretations of the standards within the framework. The inspection reports give meaning to the frameworks. This has consequences for the regulator. It becomes important what and how they write things down in their inspection reports, because this can directly impact quality improvement work in healthcare organisations. However, this research shows the impact differs between institutional settings and is especially applicable in settings where developed relations exist between associations, providers and the inspectorate.

### The design of an inspection framework matters

The design of an inspection framework influences the opportunity to explain things during an inspection. Healthcare providers indicated that they want to show why they have organised quality in specific ways and that they have given this thought. Within elderly care, this dialogue is part of the inspection: the inspector asks questions about care provision arrangements. The design of the elderly care framework, with its more open standards and gradual assessment, seems to promote this dialogue. Experiences with this new approach to inspecting were positive and could contribute to an improved relation between the inspector and the professionals. As previous research suggests, this could facilitate an open and honest conversation about the organisation’s performance.^7^ Additionally, open standards in a framework that enable dialogue could contribute to a feeling that inspections and assessments are fair as there is an opportunity to explain oneself.^27^ Procedural justice has been associated with improved compliance whereas injustice with declining compliance to standards.^28^ Finally, there seems to be a relation between the design and purpose of the framework. Strict dichotomous standards invite a more repressive use, while open standards invite dialogue and are focused more on learning and improvement.

## Conclusion

Our findings highlight that publication of inspection frameworks might result in quality improvement work and in particular contexts could be used as a regulatory strategy to target quality improvement in a healthcare sector. When using publication of inspection frameworks as a strategy to incentivise quality improvement, regulators should pay attention to the institutionalised quality routines within a sector and take this into account when designing inspection frameworks. When the purpose is to stimulate learning and improving in a sector, it is important to consider whether the institutional infrastructure of the sector, and their relationship with that sector, permits a learning and improving approach using open standards. For some sectors or themes, a public inspection framework may evoke a sector to improve on quality policy, while in other cases the framework and subsequent round of inspections have little effect on the quality and safety of the sector. In that case, regulators should not only focus on (thematic) inspection frameworks, but also look more broadly on the development of quality improvement initiatives in a sector. Ultimately, in using the inspection frameworks, attention should be paid to coordination and the relation between the design of an inspection framework, what the regulator intends to achieve with it and the possibilities of a sector.

## Data Availability

No data are available. The data generated in this study are confidential interview transcripts that are not available for sharing.
