# Supplementary material for: Publication of inspection frameworks: a qualitative study exploring the impact on quality improvement and regulation in three healthcare settings
Source: BMJ Qual Saf. 2020 Dec 2;30(10):804–11. doi: 10.1136/bmjqs-2020-011337 (PMC8461449; doi:10.1136/bmjqs-2020-011337)
Supplement: Supplementary data [file bmjqs-2020-011337supp001.pdf]

Appendix A. Topic list interviews inspection frameworks

(1) Knowledge of and attitude towards frameworks
- Awareness of the inspection frameworks, how did people become aware of the framework
- Accessibility and comprehensibility of the inspection framework
- Agreement with the included standards in the framework

(2) Use of the framework in practice
- Internal and external mechanisms that contribute to the adoption and use of an inspection framework
- Experiences with the framework during inspections

(3) Consequences/influence on compliance to norms and quality work
- In what ways does the organization steer quality processes and what role does an inspection framework and the healthcare inspectorate has in this
- Consequences of publication on healthcare professional/provider
- Consequences of publication on inspector and inspections
- Role of characteristics of the inspection framework, such as open/closed standards, understandability, accessibility

(4) Interaction between stakeholders and inspectorate
- Involvement of stakeholders in development of the inspection framework
- Communication and interaction between stakeholders after publication of the framework
- Relation between the healthcare sector and stakeholders and the inspectorate and inspectors
